# Efficacy of indoor air purification in the treatment of *Artemisia* pollen‐allergic rhinitis: A randomised, double‐blind, clinical controlled trial

**DOI:** 10.1111/coa.13514

**Published:** 2020-03-25

**Authors:** Li Li, Li Zhang, Jin‐Han Mo, Yun‐Ying Li, Ji‐Yan Xia, Xiao‐Bing Bai, Pei‐Fang Xie, Jing‐Yi Liang, Zi‐Feng Yang, Qiao‐Yan Chen

**Affiliations:** ^1^ The First Hospital of Yulin The Second Affiliated Hospital Yanan University Yan’an China; ^2^ Beijing Key Laboratory of Indoor Air Quality Evaluation and Control Tsinghua University Beijing China; ^3^ Guangdong Provincial Hospital of Chinese Medicine The Second Affiliated Hospital Guangzhou University of Chinese Medicine Guangzhou China; ^4^ State Key Laboratory of Respiratory Diseases Guangzhou Institute of Respiratory Disease National Clinical Centre of Respiratory Disease The First Affiliated Hospital Guangzhou Medical University Guangzhou China; ^5^ State Key Laboratory of Quality Research in Chinese Medicines Macau University of Science and Technology Macau China

**Keywords:** allergy, quality of life, randomised controlled trials, rhinitis, sleep

## Abstract

**Objectives:**

To evaluate the clinical efficacy of a high‐efficiency air purifier in patients with allergic rhinitis.

**Design:**

We conducted a randomised, double‐blind, clinical controlled trial with active and inactive versions of an air purifier. Our study included patients with allergic rhinitis who were sensitive to *Artemisia* pollen and treatment of the indoor environment using air filtration at night. We evaluated the clinical efficacy of indoor air filtration during the *Artemisia* pollen scattering season in Yulin City in Shanxi Province, China.

**Setting:**

The First Hospital of Yulin (Yulin City, Shanxi Province, China).

**Participants:**

A total of 90 patients with allergic rhinitis who were sensitive to allergens of *Artemisia* pollen were randomly assigned to one of two groups in equal numbers.

**Main outcome measures:**

The primary outcome measure was the difference in visual analogue scale scores from baseline. Secondary outcomes were changes from baseline in nasal symptoms, allergy symptom scores, responses to the Rhinoconjunctivitis Quality of Life Questionnaire, Epworth Sleepiness Scale scores and tolerability scores for the air purifier.

**Results:**

Based on the allergy symptom score, we found significant differences in rhinitis symptoms between the groups who used the active versus the inactive air purifier.

**Conclusions:**

The results of our investigation demonstrated the health benefits of particle filtration.

**Keypoints**

- In theory, avoiding exposure to allergens could lead to recovery without treatment. In this study, patients used an air purifier to avoid allergens as much as possible at night, but they were still exposed to allergens during the day. Though the time of allergen exposure was reduced, the allergens the patients were exposed to during the day would trigger allergic reactions.
- *Artemisia* pollen can be filtered with an air purifier.
- For some patients with allergic rhinitis, seasonal migration may alleviate allergy symptoms, but this is an unrealistic option for most.
- We sought to identify a convenient, economical and practical way to avoid or mitigate allergen exposure.

## 1 INTRODUCTION

### 1.1 Background

*Artemisia* pollen is one of the most common causes of pollinosis in many parts of the world.^1^, ^2^, ^3^, ^4^, ^5^ Pollen concentrations fluctuate daily and by region. Theoretically, avoiding exposure to allergens could lead to recovery without treatment. While seasonal migration may alleviate allergy symptoms for some patients with AR, this is an unrealistic option for most. In Japan, a nationwide map of daily pollen concentrations is published to help patients with AR avoid areas with high pollen concentrations.^6^ When outdoor pollen levels are high, patients with AR are advised to stay indoors. Although particle filtration can modestly reduce the adverse outcomes of allergy and asthma in homes with pets,^7^ the findings of previous related studies varied because of differences in observation times and the enrolled participants.^8^, ^9^, ^10^, ^11^

Exposure to *Artemisia* pollen may cause an attack or exacerbation of allergic rhinitis (AR) among people who are sensitive to the allergens of the pollen. The diameter of *Artemisia* pollen ranges from 19 to 25 μm, which means it can be filtered with an air purifier.^12^ Thus, treatment with air purifiers is a viable option to limit exposure and thereby improve clinical outcomes. We sought to identify a convenient, economical and practical way to avoid or mitigate allergen exposure. Therefore, in this study, we aimed to determine the effect of an indoor air environment with low or minimal allergen density via indoor air purification at night to reduce the exposure time to allergens in the home.

### 1.2 Objectives

We aimed to assess the clinical efficacy of indoor air filtration during the *Artemisia* pollen scattering season in Yulin City, Shanxi Province, China. Primarily, we wished to determine whether artificially reducing or eliminating indoor pollen concentrations could help minimise exposure to *Artemisia* pollen during the pollen scattering season and whether such treatment can have beneficial clinical effects in patients with AR.

## 2 MATERIALS AND METHODS

### 2.1 Ethical considerations

This clinical trial was reviewed, and ethical approval for the study protocol was granted by the Ethics Committee of the First Hospital of Yulin (number 201601). All patients were required to sign consent forms to participate in the study. Written informed consent was obtained from each participant. All personal identifying information was protected.

### 2.2 Study design and participants

The study protocol and details of the physiological and biochemical assessments undertaken have been described previously.^13^ This study was implemented in Yulin City from June 2016 to September 2018. We enrolled patients with AR, based on the definition outlined by ARIA,^14^ and those who showed sensitivity to the allergens of *Artemisia* pollen in the recruitment radioallergosorbent test (RAST). They were referred to the study team by physicians of the First Hospital of Yulin. All patients lived in Yulin. In this randomised, double‐blind, placebo‐controlled clinical trial, we tested active and placebo versions of an air purifier. The air purifiers were equipped with monitors that could measure the number of hours of operation. Enrolled patients were randomly assigned to one of two groups in equal numbers. All patients underwent a 4‐week treatment period and a 4‐week observation period. Patient evaluation was conducted at baseline (day 0) and on days 7, 14, 21 and 28 (Table 1). Specific tests conducted at each follow‐up session are outlined in Table 1.

**Table 1:** Schedule of patient evaluation

Outcome measures	Screening stage	Remedial period				Observation period
	Baseline	Day 7	Day 14	Day 21	Day 28	Day 56
Nasal symptoms	X	X	X	X	X	
Allergy symptom score	X	X	X	X	X	
Visual analogue scale score	X	X	X	X	X	
Rhinoconjunctivitis Quality of Life Questionnaire	X	X	X	X	X	
Epworth Sleepiness Scale score	X	X	X	X	X	
Tolerability of the air purifier		X	X	X	X	
Treatment compliance		X	X	X	X	
Safety assessment		X	X	X	X	X

At patient enrolment, collection of specimens and data was undertaken at the Outpatient Department of the First Hospital of Yulin. Biochemical examinations were carried out by King Med Diagnostics (Guangzhou, China). The data were analysed at the University of Toronto (Canada).

#### 2.2.1 Enrolment

We recruited, screened and enrolled or excluded patients on the basis of specific criteria. Patients were clearly informed of the study aims and procedures, as well as their right to discontinue participation in this trial at any time. After patients signed consent forms, the research staff randomly assigned recruited patients to the treatment or control group. Inclusion and exclusion criteria, as well as criteria for participant rejection or termination, are presented in Tables 2 and 3.

**Table 2:** Inclusion and exclusion criteria

Inclusion criteria	Exclusion criteria
Confirmed allergic rhinitis	Mental disorders, asthma
Sensitive to *Artemisia* pollen allergens	Current or recent serious systemic disease. Systemic disease that the researchers considered would interfere with the study.
Aged 18‐65 y	Age under 18 y or over 65 y
Provided informed consent and volunteered to participate in this clinical trial	Not cooperative during examinations
Completed the case report form and other records	Employment changes leading to a possible loss to follow‐up
	Dysgnosia or behavioural disorders
	The following conditions: nasal polyps, chronic sinusitis, severe nasal deviation, rhinitis medicamentosa, primary sleep disorders (>1 night/wk), obstructive sleep apnoea, upper respiratory infection within 2 wk prior to enrolment or poorly controlled asthma
	Pregnant or may become pregnant, or lactating with a positive urine pregnancy test
	Drug abuse within the past 3 y
	Must sleep in a different bed more than six times in 3 wk or for more than three consecutive nights
	Smoked within the past 1 y
	Sensitive to indoor allergens such as dust mites and pet dander
	Other reasons, at the investigator's discretion
	Refusal to continue the trial because of a poor curative effect
	Refusal to continue the trial for an unspecified reason
	Loss to follow‐up because of a change of address or telephone number
	Loss to follow‐up because of personal reasons

**Table 3:** Rejection and termination criteria

Rejection criteria	Termination criteria
(1) Did not meet the inclusion criteria	(1) Symptoms (eg sneezing, runny nose, nasal obstruction or nose itching) that become severe
(2) Withdrew written informed consent	(2) Occurrence of a serious event
(3) Did not receive follow‐up care after selection for the trial	(3) Other health reasons sufficient to halt participation in the study.
(4) Violated the terms of the trial (eg improper use of air purifiers, leading to effects that cannot be evaluated)	(4) Not cooperated with the examination

#### 2.2.2 Interventions in the treatment and control groups

An Atmosphere^®^ air purifier (Amway, China) was placed in the participants’ bedrooms. The air purifiers provided to participants in the treatment group contained an Atmosphere® HEPA (model number 101076CH) two‐way filter. This filter has an airflow velocity of 100‐200 cubic feet/min and a filtration rate of 6000‐12 000 cubic feet/hour. This purifier produces 4‐8 air changes per hour in a typical bedroom measuring 15 × 12 × 8 feet. The air purifiers provided to participants in the control group contained a placebo filter, which also had a two‐way design with an airflow velocity of 100‐200 cubic feet/min. Instructions were given to keep the purifier running continuously even if participants left the bedroom. Participants were required to remain in their bedroom at night for 4 weeks, that is, participants were to remain in their bedroom for >8 hours per day.

#### 2.2.3 Concomitant care and intervention

During the treatment and follow‐up periods, participants were prohibited from taking medications such as antihistamines (p.o., i.n.), corticosteroids (i.n.), decongestants (i.n.) or leukotriene receptor antagonists (p.o.). Only patients with severe symptoms with major effects, such as a severe decline in the patient's sleep quality or the quality of work and daily life, were treated with anti‐allergic agents, since better medical treatment for these patients was more important than adherence to the study protocol. The type of medication and dose was recorded in a diary kept by the participants. For other complicated chronic diseases, patients were asked to continue taking their routine medications and other therapies. The research staff recorded all details of diseases, medications and therapies in the case reports. Patients declared the time for which they actually used the air purifier everyday.

### 2.3 Variables

Differences in symptom severity and quality of life (QoL) served as the primary outcome measures and were assessed using a visual analogue scale (VAS). The 7‐point VAS included scores ranging from 1 (no symptom) to 7 (worst‐ever symptoms). Secondary outcomes were changes in nasal symptoms and allergy symptoms, as well as scores on the Rhinoconjunctivitis Quality of Life Questionnaire (RQLQ) and Epworth Sleepiness Scale, and tolerability scores for the Atmosphere^®^ air purifier. Evaluated nasal symptoms included swelling of the turbinates, graded as 1 (mild), 2 (moderate) or 3 (severe). Allergy symptom scores ranged from 0 to 3, with symptoms graded as 0 (no symptoms), 1 (slight symptoms), 2 (moderate symptoms) and 3 (severe symptoms). Symptoms to be graded included congestion, sneezing, nasal itchiness, rhinorrhea, eye itchiness, ear/palate itchiness, eye redness and tearing. The RQLQ contains 28 questions covering seven topics (daily life activities, sleep, non‐eye/nasal symptoms, practical problems, nasal symptoms, eye symptoms and emotional status), each with scores ranging from 0 (none) to 6 (very often/always). The Epworth Sleepiness Score consists of eight questions evaluating sleepiness status, which are answered using scores from 0 (none) to 3 (probably). The tolerability score for the Atmosphere^®^ air purifier was based on five questions for tolerability of this air purifier. Answers were graded from 1 (completely intolerable) to 5 (completely tolerable).

### 2.4 Data collection and management

Research staff were responsible for the data collection. A third party set up the study database and programme settings and also implemented monitoring and management of the data. All data were imported in duplicate into an electronic database by two assistants. Identified input errors were corrected to ensure that there were no differences in the database. The statistical manager was responsible for data organisation, coding, range checking of data values and data conversion to ensure quality of the data.

### 2.5 Data analysis

Statistical analyses were performed at the Guangzhou Institute of Respiratory Diseases using SPSS version 17.0 (SPSS Inc). Continuous variables were expressed as mean (±SD) and categorical data as n (%). All statistical inferences were determined using two‐sided tests. A significance level of 0.05 with 95% confidence intervals was used to measure the uncertainty of the estimates. Baseline data analyses (two sets) included demographic indicators, history of AR and smoking status of the participants and their family members. Measurement data were compared using a Student t test. Pearson's chi‐squared test was used to compare the groups using active and inactive air purifiers. Effects of interventions on the outcome indicators were evaluated by logistic regression models that included the VAS score, RQLQ score, allergy symptom score, Epworth Sleepiness Scale score and tolerability score for the Atmosphere^®^ air purifier.

## 3 RESULTS

A total of 90 participants completed the intervention. All participants had a history of *Artemisia* pollen‐induced rhinitis, and those with severe AR symptoms were not included in this study. Three participants dropped out of the study because of an inability to comply with the study protocol. The data from patients’ self‐management records and the machine operation records both showed good patient compliance for remaining in their bedroom for >8 hours per day. A total of 45 participants each were randomised to the active and inactive air purifier groups. Medical and demographic characteristics of the study groups at baseline are outlined in Table 4. The average age of the treatment and control groups was 35.53 and 36.11 years, respectively. There were 24 (53%) women and 21 (47%) men in the active air purifier group and 26 (58%) women and 19 (42%) men in the inactive air purifier group. All participants were non‐smokers, but family members of five participants (11%) in the treatment group smoked; family members of three participants (7%) in the control group were smokers. There were no significant differences between the active and inactive air purifier groups for any medical or demographic variables (Table 4).

**Table 4:** Patient characteristics

	Intervention	Control	*P* value
Number of patients	45	45	
Age	35.5 (±8.2)	36.1 (±9.2)	.364
History of allergic rhinitis (y)	8.6 (±5.4)	7.3 (±5.2)	.310
Gender			
Male	21 (47%)	19 (42%)	.671
Female	24 (53%)	26 (58%)	
Smoking status of patient			
Yes	0	0	‐
No	45 (100%)	45 (100%)	
Smoking status of patient's family			
Yes	5 (11%)	3 (7%)	.459
No	40 (89%)	42 (93%)	

Note

Data are n (%) or mean (±SD).

The allergy symptom scores showed significant differences between the active and inactive air purifier groups with respect to rhinitis symptoms, with a p value of 0.004. No significant differences were detected between the groups for measures on the RQLQ (Table 5).

**Table 5:** Effect of the intervention on outcome indicators

	Intervention			Control			Relative change^a^ (95% CI; *P* value)
	Baseline	Endline	Mean difference	Baseline	Endline	Mean difference	
VAS	2.4	3.7	1.3 (1.9)	2.4	3.5	1.1 (1.8)	0.2 (−0.4, 0.9); .483
RQLQ	96.9	58.7	−38.2 (36.4)	101.3	62.7	−38.6 (52.4)	0.4 (−15.2, 14.4); .959
Daily life activities	10.8	7.0	−3.8 (4.7)	10.9	7.7	−3.2 (6.0)	‐0.6 (−1.3, 2.6); .502
Sleep	9.5	5.9	−3.6 (4.4)	10.7	6.4	−4.3 (7.0)	0.7 (−3.0, 1.6); .531
Non‐eye/nasal symptoms	20.4	13.5	−6.9 (10.1)	21.5	14.4	−7.1 (14.5)	0.2 (−4.6, 4.0); .892
Practical problems	12.8	7.3	−5.5 (5.4)	13.5	8.3	−5.2 (5.8)	‐0.3 (−1.6, 2.2); .779
Nasal symptoms	16.5	9.2	−7.3 (6.3)	16.8	10.0	−6.8 (8.0)	‐0.5 (−1.8, 2.9); .635
Eye symptoms	14.2	7.8	−6.4 (6.7)	13.7	7.7	−6.0 (7.9)	‐0.4 (−2.1, 2.8); .788
Emotional status	12.7	8.1	−4.6 (5.8)	14.2	8.3	−5.9 (8.2)	1.3 (−3.9, 1.2); .290
Allergy symptom score	18.3	9.3	−9.0 (6.0)	17.1	11.4	−5.7 (7.4)	‐3.3 (1.1, 5.5); .004
Epworth Sleepiness Scale score	10.9	8.3	−2.6 (6.9)	12.2	9.6	−2.6 (6.1)	<0.01 (−2.7, 2.5); .931
Tolerability score for the Atmosphere air purifier	22.6	19.3	−3.3 (7.0)	21.4	18.7	−2.7 (5.4)	‐0.6 (−1.9, 3.0); .677

Abbreviations: RQLQ, Rhinoconjunctivitis Quality of Life Questionnaire; VAS, visual analogue scale.

Data are mean or mean (±SD).

^a^ Relative change refers to mean difference between the intervention and control groups.

**Figure 1:**
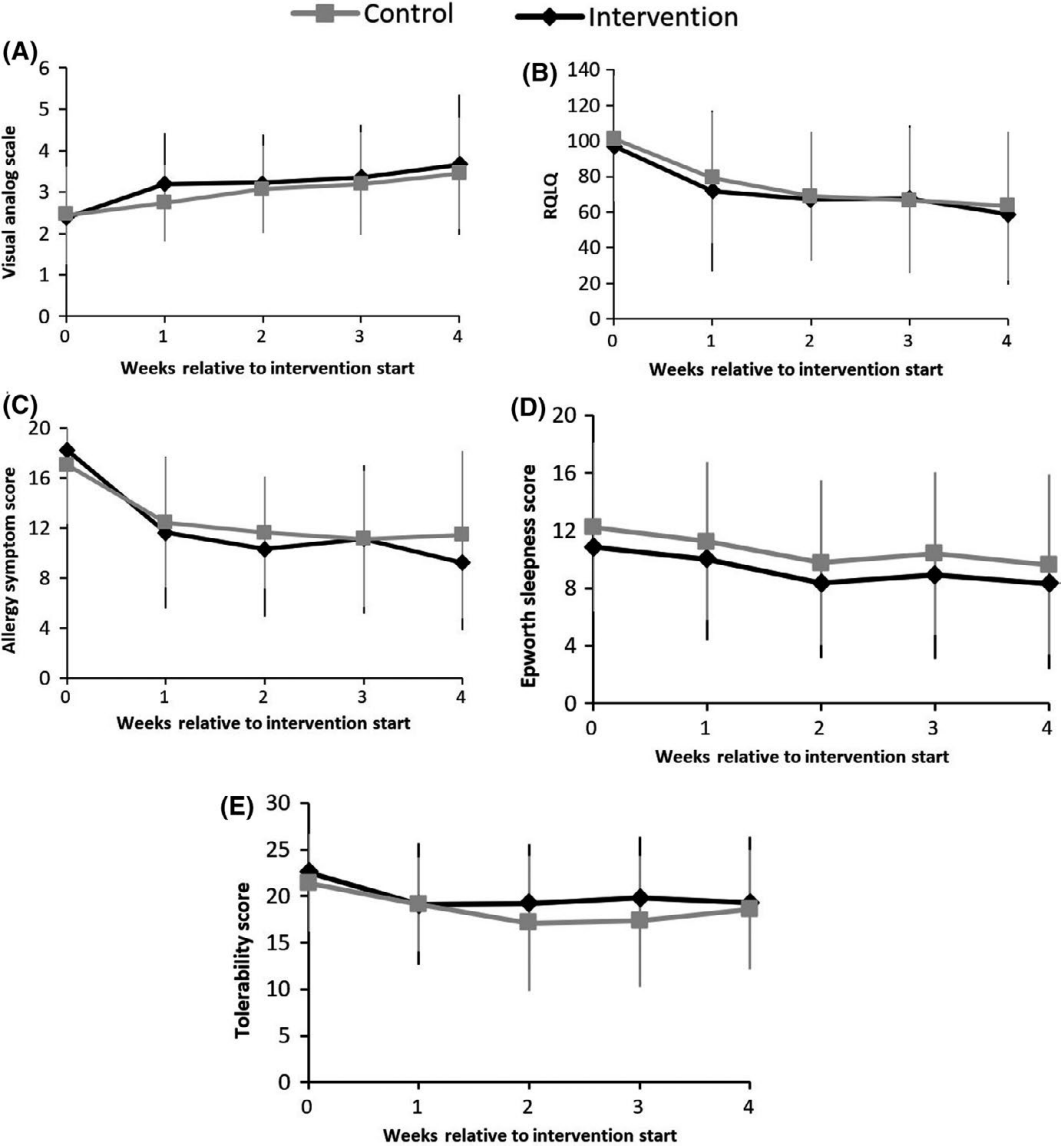
Weekly change in outcomes after intervention. RQLQ: Rhinoconjunctivitis Quality of Life Questionnaire

The RQLQ contains 28 questions covering seven topics, and the cumulative score for the seven topics is considered the score for the general status indicators. A line chart revealed that the cumulative score for the general status indicators showed a significant decline in both groups; furthermore, the score for each item declined gradually each week. The cumulative score in the active air purifier group fell from a baseline value of 96.89 to 58.67. The cumulative score in the control group fell from a baseline value of 101.27 to 63.45 (Table 5, Figure 1).

The line chart also revealed that the following scores decreased progressively each week from baseline to the end of the intervention: nasal symptom score, allergy symptom score (VAS), Epworth Sleepiness Scale score and tolerability score for the Atmosphere^®^ air purifier. The nasal symptom data trend line cut high and low in both directions between the active and inactive air purifier groups. The sleepiness data trend decreased in steps between the two groups. The trend of the tolerability score for the Atmosphere^®^ air purifier also decreased with no lines crossing between the groups, but the range ability during the second week in the control group was greater than that in the treatment group. The data trend in the inactive air purifier group was ascending at the end of intervention. VAS scores showed progressive weekly increases; scores were higher in the intervention group than in the controls (Table 5, Figure 1).

## 4 DISCUSSION

### 4.1 Synopsis of the key findings

*Artemisia* is a rare annual, biennial or perennial herbaceous bush that mostly grows on slopes, in the wilderness or by the roadside. With the accelerating urbanisation in China, desertification has become increasingly predominant in parts of the country. Severe soil erosion by wind is the main pattern of desertification in China.^15^ The most effective and fundamental measure to prevent soil erosion is to improve the surface vegetation cover. As an adaptable, drought‐resistant, sand‐fixing plant, *Artemisia* is the primary type of vegetation used for desertification control. Yulin City is a typical area experiencing desertification. Yulin is located in the far northern part of Shanxi Province, at the junction of the Loess Plateau and Mu Us Desert, the transition zone between the Loess and Inner Mongolian plateaus, a region where the vegetation coverage is very low. Experimental afforestation using *Artemisia* has been conducted in the Yulin area by the government of China since the 1950s.^16^ With the accelerated urbanisation and increasingly greater *Artemisia* vegetation in the region, the number of patients with AR in this part of the country has progressively increased.

We chose individuals with AR who were sensitive to *Artemisia* pollen as the focus of this study for three main reasons. First, *Artemisia* pollen can be filtered using an air purifier.^12^ Second, *Artemisia* pollen is extremely common; pollen counts can be obtained for each province of mainland China. *Artemisia* is the primary outdoor allergen in China.^17^ Third, it has been proposed that an air purifier is likely to be of benefit against *Artemisia* pollen because this outdoor allergen is common inside the home in many regions of China. Fourth, the incidence of *Artemisia* pollen allergy is increasing together with urbanisation in Yulin City, a typical area undergoing desertification in China.

Using a double‐blind, placebo‐controlled protocol, we sought to evaluate the effects of a room air purifier in patients with sensitivity to *Artemisia* pollen. We found that rhinitis symptoms improved significantly after the intervention. Intervention studies conducted in the homes of patients with allergy or asthma who were supplied with filtered air to the areas where they slept have reported improvements in some assessed health outcomes. In our study, participants in the intervention group used an air purifier to deliver filtered air to their sleeping areas, such as the bedroom. Tolerability scores for the Atmosphere^®^ air purifier in the treatment group were lower than those in the control group. This could be because rhinitis symptoms improved, leading to improved tolerance. Our findings also showed that the air purifier had little impact on sleep at night, even when placed in the bedroom.

### 4.2 Comparison with other studies and clinical applicability

Morris and co‐workers conducted research into 1‐week, nocturnal, indoor air purification treatment in patients who were allergic to ragweed. Those authors recommended that patients with seasonal AR use air purification devices during the ragweed pollen season.^9^ Stillerman et al investigated patients with perennial AR who underwent 12‐week air purification treatment. During this time, nasal congestion, sneezing, runny nose, itchy eyes, tearing and other symptoms improved, as did QoL. Therefore, those authors suggested that effectively reducing allergen exposure has clinical value.^11^ Those studies suggest that air purification devices used at night have clinical benefit in patients with AR. In the present study, the results of the Allergy symptom score showed that rhinitis symptoms had significantly improved. Our findings suggest that reducing allergic desensitisation by limiting exposure to allergens at night could have clinical applications.

Allergen exposure is considered to be an important risk factor for allergic respiratory disease.^18^ Bronchoprovocation experiments have proven that allergens could induce bronchospasm, eosinophilic airway inflammation and prolonged increases in bronchial hyperreactivity, indicating that allergen exposure was related to asthma.^19^ These findings indicate that allergic patients should reduce allergen exposure in their houses as part of the management of asthma and AR.^20^ However, the findings did not clarify why the asthma symptoms did not completely resolve with the rhinitis. This study showed that the air purifier may be effective for AR, but we did not enrol participants who had asthma. The benefits of air purifiers for patients with asthma who were sensitive to *Artemisia* pollen and the effect of the environment on the progression of allergic disease may deserve additional research.

### 4.3 Limitations of the study

In this pilot study, no other metrics (outside of the allergy symptom score) were different between the two groups. The number of patients was relatively small; therefore, further studies with a larger number of patients are needed to confirm our findings. It is also possible that more aggressive environmental control measures would produce a greater effect. Furthermore, the use of additional air purifiers in other areas of the home or workplace could further reduce allergen exposure and thereby reduce allergy symptoms. We did not include this measure because our intent in this study was to assess the effect of a relatively simple approach that can be applied by most patients, namely use of an air purifier in the bedroom.

If patients had AR symptoms, medications could provide them relief, but some patients did not show complete remission. Even though patients had light AR symptoms, they did not always take medication. Before they enrolled in the study, they would have hesitation period. They had been informed that the air purifier was a kind of replacement therapy, and they received anti‐allergic agents only when they were treated with severe symptoms. For some patients with AR, seasonal migration may alleviate allergy symptoms, but this is an unrealistic option for most. When outdoor pollen levels were high, the morbidity caused by AR increased. We aimed to determine the effect of an indoor air environment with low or minimal allergen density via indoor air purification at night, in reducing the exposure time to allergens in the home. Therefore, in this study, we did not enrol patients with poorly controlled AR.

## 5 CONCLUSION

We investigated the clinical effect of air purification among participants with AR who were sensitive to the allergens of *Artemisia* pollen. The present findings demonstrated the health benefits of particle filtration. Filtration may be modestly effective in reducing adverse outcomes of AR, particularly in homes with *Artemisia* pollen, such as areas with desertification. Our study also suggested that filtration of air in the sleeping areas of individuals with allergies may be effective in improving health.

## CONFLICTS OF INTEREST

The authors declare that they have no competing interests.
